# Is the Infection of the SARS-CoV-2 Delta Variant Associated With the Outcomes of COVID-19 Patients?

**DOI:** 10.3389/fmed.2021.780611

**Published:** 2021-12-09

**Authors:** Gunadi, Mohamad Saifudin Hakim, Hendra Wibawa, Marcellus, Vivi Setiawaty, Slamet, Ika Trisnawati, Endah Supriyati, Riat El Khair, Kristy Iskandar, Afiahayati, Siswanto, Irene, Nungki Anggorowati, Edwin Widyanto Daniwijaya, Dwi Aris Agung Nugrahaningsih, Yunika Puspadewi, Dyah Ayu Puspitarani, Irene Tania, Khanza Adzkia Vujira, Muhammad Buston Ardlyamustaqim, Gita Christy Gabriela, Laudria Stella Eryvinka, Bunga Citta Nirmala, Esensi Tarian Geometri, Abirafdi Amajida Darutama, Anisa Adityarini Kuswandani, Lestari, Sri Handayani Irianingsih, Siti Khoiriyah, Ina Lestari, Nur Rahmi Ananda, Eggi Arguni, Titik Nuryastuti, Tri Wibawa

**Affiliations:** ^1^Pediatric Surgery Division, Department of Surgery/Genetics Working Group, Faculty of Medicine, Public Health and Nursing, Universitas Gadjah Mada, Yogyakarta, Indonesia; ^2^Department of Microbiology, Faculty of Medicine, Public Health and Nursing, Universitas Gadjah Mada, Yogyakarta, Indonesia; ^3^Disease Investigation Center, Ministry of Agriculture Indonesia, Yogyakarta, Indonesia; ^4^National Institute of Health Research and Development, Ministry of Health, Jakarta, Indonesia; ^5^Pulmonology Division, Department of Internal Medicine, Faculty of Medicine, Public Health and Nursing, Universitas Gadjah Mada/Dr. Sardjito Hospital, Yogyakarta, Indonesia; ^6^Centre of Tropical Medicine, Faculty of Medicine, Public Health and Nursing, Universitas Gadjah Mada, Yogyakarta, Indonesia; ^7^Department of Clinical Pathology and Laboratory Medicine, Faculty of Medicine, Public Health and Nursing, Universitas Gadjah Mada/Dr. Sardjito Hospital, Yogyakarta, Indonesia; ^8^Department of Child Health, Faculty of Medicine, Public Health and Nursing, Universitas Gadjah Mada, Yogyakarta, Indonesia; ^9^Department of Computer Science and Electronics Faculty of Mathematics and Natural Sciences, Universitas Gadjah Mada, Yogyakarta, Indonesia; ^10^Department of Physiology, Faculty of Medicine, Public Health and Nursing, Universitas Gadjah Mada/UGM Academic Hospital, Yogyakarta, Indonesia; ^11^Balai Besar Teknik Kesehatan Lingkungan dan Pengendalian Penyakit, Yogyakarta, Indonesia; ^12^Department of Anatomical Pathology/Genetics Working Group, Faculty of Medicine, Public Health and Nursing, Universitas Gadjah Mada, Yogyakarta, Indonesia; ^13^Department of Pharmacology and Therapy/Genetics Working Group, Faculty of Medicine, Public Health and Nursing, Universitas Gadjah Mada, Yogyakarta, Indonesia; ^14^RSUD Dr. Loekmono Hadi, Kudus, Indonesia

**Keywords:** comorbidity, Ct value, delta variant, hospitalization, mortality, SARS-CoV-2, viral load, whole genome sequencing

## Abstract

**Background:** Severe acute respiratory syndrome Coronavirus 2 (SARS-CoV-2) Delta variant (B.1.617.2) has been responsible for the current increase in Coronavirus disease 2019 (COVID-19) infectivity rate worldwide. We compared the impact of the Delta variant and non-Delta variant on the COVID-19 outcomes in patients from Yogyakarta and Central Java provinces, Indonesia.

**Methods:** In this cross-sectional study, we ascertained 161 patients, 69 with the Delta variant and 92 with the non-Delta variant. The Illumina MiSeq next-generation sequencer was used to perform the whole-genome sequences of SARS-CoV-2.

**Results:** The mean age of patients with the Delta variant and the non-Delta variant was 27.3 ± 20.0 and 43.0 ± 20.9 (*p* = 3 × 10^−6^). The patients with Delta variant consisted of 23 males and 46 females, while the patients with the non-Delta variant involved 56 males and 36 females (*p* = 0.001). The Ct value of the Delta variant (18.4 ± 2.9) was significantly lower than that of the non-Delta variant (19.5 ± 3.8) (*p* = 0.043). There was no significant difference in the hospitalization and mortality of patients with Delta and non-Delta variants (*p* = 0.80 and 0.29, respectively). None of the prognostic factors were associated with the hospitalization, except diabetes with an OR of 3.6 (95% CI = 1.02–12.5; *p* = 0.036). Moreover, the patients with the following factors have been associated with higher mortality rate than the patients without the factors: age ≥65 years, obesity, diabetes, hypertension, and cardiovascular disease with the OR of 11 (95% CI = 3.4–36; *p* = 8 × 10^−5^), 27 (95% CI = 6.1–118; *p* = 1 × 10^−5^), 15.6 (95% CI = 5.3–46; *p* = 6 × 10^−7^), 12 (95% CI = 4–35.3; *p* = 1.2 × 10^−5^), and 6.8 (95% CI = 2.1–22.1; *p* = 0.003), respectively. Multivariate analysis showed that age ≥65 years, obesity, diabetes, and hypertension were the strong prognostic factors for the mortality of COVID-19 patients with the OR of 3.6 (95% CI = 0.58–21.9; *p* = 0.028), 16.6 (95% CI = 2.5–107.1; *p* = 0.003), 5.5 (95% CI = 1.3–23.7; *p* = 0.021), and 5.8 (95% CI = 1.02–32.8; *p* = 0.047), respectively.

**Conclusions:** We show that the patients infected by the SARS-CoV-2 Delta variant have a lower Ct value than the patients infected by the non-Delta variant, implying that the Delta variant has a higher viral load, which might cause a more transmissible virus among humans. However, the Delta variant does not affect the COVID-19 outcomes in our patients. Our study also confirms that older age and comorbidity increase the mortality rate of patients with COVID-19.

## Introduction

The recent pandemic of Coronavirus disease 2019 (COVID-19) continuously causes a tremendous impact on both global health and the economy, with millions of people losing their lives. The causative agent, severe acute respiratory syndrome Coronavirus 2 (SARS-CoV-2), is believed to emerge from bats as the natural reservoir of various strains of coronaviruses (CoV), including SARS-CoV-2 and the former SARS-CoV (1). Many SARS-CoV-2 infections in this ongoing pandemic have provided space and opportunity for continuous mutation and evolution, giving rise to novel variants with increased fitness and, thus, a significant impact on public health (2).

The SARS-CoV-2 variants of concern (VOC), including Alpha, Beta, Gamma, and Delta, have attracted public health authorities due to its capability on higher transmission; the possibility of affecting COVID-19 severity; and the impact of the effectiveness of public health measures, diagnosis, treatment, and vaccines (3–5). Among those VOC, the SARS-CoV-2 Delta variant (B.1.617.2) has been responsible for the current increase in COVID-19 infectivity rate worldwide, including Indonesia (6–10). The Delta variant is approximately two times more transmissible than the previous variants (11). The higher fitness and transmission capacity of the Delta variant is partly attributed to the notable mutations in the spike (S) region, including P681R and L452R, leading to a higher affinity of angiotensin-converting enzyme 2 (ACE-2) attachment (12, 13).

Currently, the Delta variant has been the most frequent variant circulating globally (14). Indeed, national genomic surveillance of SARS-CoV-2 variants has identified the Delta variant as the most dominant circulating variant in Indonesia (97.8%), followed by Alpha (1.7%) and Beta variants (0.5%) (10). Noteworthy, the Delta variant has been associated with a higher risk of hospitalization, more severe outcomes, admission of ICU, and mortality than other variants (15–17).

Several prognostic factors have been associated with COVID-19 illness (18–20). However, our knowledge about the role of the Delta variant on the COVID-19 outcomes is still very limited (15, 21). In this study, we compared the impact of the Delta variant and non-Delta variant infections on the COVID-19 outcomes, i.e., hospitalization and mortality, in patients from Yogyakarta and Central Java provinces, Indonesia.

## Materials and Methods

### Patients

This cross-sectional study ascertained 161 patients with COVID-19 (79 men and 82 women) from Yogyakarta and Central Java provinces. The patients were ascertained in this study if the PCR's Ct value was ≤25 according to our previous studies (22–24). The diagnostic criteria of COVID-19 were determined using PCR. The PCR was performed for patients with clinical manifestations of COVID-19 or close contact with the confirmed COVID-19 case.

Moreover, some patients (45/69, 65.2%) infected by the Delta variant were confirmed in clusters, while others (24/69, 34.8%) were not. The outcomes of patients with COVID-19 were hospitalization and mortality.

The sample size was determined using the cross-sectional design formula: type I error rate (α) of 0.05, power of the study (1–β) of 0.63, the odds ratio of Delta patients for hospitalization of 1.85, and proportion of hospitalization for non-Delta patients of 0.19 (15). The calculated total sample size was 160.

### Prognostic Factors

According to previous studies, we associated the following prognostic factors with the hospitalization and mortality of patients with COVID-19: sex; age; comorbidities, including obesity, diabetes, hypertension, cardiovascular disease, and chronic kidney disease; and smoking (18–20).

### Sample Collection

All samples were collected from either outpatient or hospitalized patients with COVID-19 from May 2020 to June 2021 from Yogyakarta and Central Java provinces. We diagnosed the first patient infected with a non-Delta and Delta variant on May 16, 2020, and May 25, 2021, respectively. Samples were collected from nasopharyngeal swabs by using viral transport media. Subsequently, the samples were sent to our institution for PCR.

### Severe Acute Respiratory Syndrome Coronavirus 2 Whole-Genome Sequencing

According to our previous studies, SARS-CoV-2 WGS was performed for all samples with PCR's Ct value of ≤25 (22–24). First, single-stranded cDNA was synthesized from RNA extracted from the viral transport medium of patients with COVID-19 using SuperScript™ III First-Strand Synthesis System (Thermo Fisher Scientific, MA, United States). Then, the second strand was synthesized using COVID-19 ARTIC v3 primer pool design by SARS-CoV-2 ARTIC Network using Phusion™ High-Fidelity DNA Polymerase (Thermo Fisher Scientific, MA, United States). The library preparations were performed using the Illumina DNA Prep (Illumina, California, United States). The Illumina MiSeq next-generation sequencer was used to perform the whole-genome sequences of SARS-CoV-2. The assembly of our sample genomes was mapped into the reference genome from Wuhan, China (hCoV-19/Wuhan/Hu-1/2019, GenBank accession number: NC_045512.2) using Burrow–Wheeler Aligner (BWA) algorithm embedded in UGENE v. 1.30 (25).

### Phylogenetic Study

We used a dataset of 250 available SARS-CoV-2 genomes extracted from GISAID from our region and others (Acknowledgment Table is provided in Supplementary Table 1) to reconstruct the phylogenetic tree. Multiple nucleotide sequence alignment was performed using the MAFFT program (https://mafft.cbrc.jp/alignment/server/). We used the neighbor-joining statistical method with 1,000 bootstrap replications (26, 27) to construct a phylogenetic tree from 29.420 nucleotide length of the open reading frame (ORF) of SARS-CoV-2, followed by computation of the evolutionary distances, and model the rate variation among sites by the Kimura 2-parameter method and the gamma distribution with estimated shape parameter (α) for the dataset, respectively (28). We used the DAMBE version 7 (29) to calculate the estimation of the gamma distribution, MEGA version 10 (MEGA X) (30), for phylogenetic reconstruction, and FigTree to visualize the Newick tree output from MEGA X.

### Statistical Analysis

We presented data as mean ± SD and frequency (percentage). The normality of the continuous variables was evaluated using the Kolmogorov–Smirnov test. Missing or incomplete data were excluded from the final analysis. Chi-square or Fisher's exact tests with 95% confidence interval (CI) were used to find any significant association between independent variables and COVID-19 outcomes. Next, multivariate analysis was performed directly using a logistic regression test. We included all variables in multivariate analysis because those prognostic factors have been associated with the outcomes of patients with COVID-19 (18–20). The *p*-value of <0.05 was considered significant. All statistical analyses were performed using the IBM Statistical Package for the Social Sciences (SPSS) version 21 (Chicago, United States).

### Ethical Approval

The Ethics Committee of the Faculty of Medicine, Public Health and Nursing, Universitas Gadjah Mada/Dr. Sardjito Hospital approved our study (KE/FK/0563/EC/2020).

## Results

### Phylogenetic Analysis

**Figure 1:**
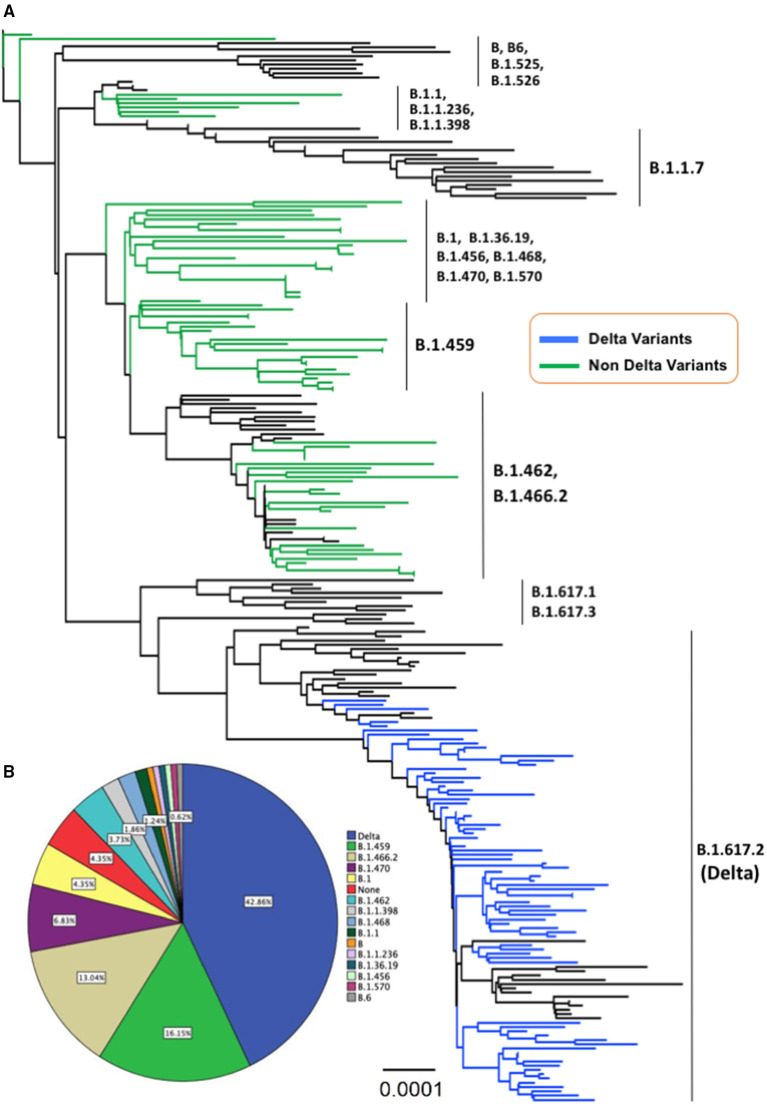
**(A)** The evolutionary history was inferred using the neighbor-joining method conducted in MEGA-X. The evolutionary distances were computed using the Kimura 2-parameter method with 1,000 bootstrap replication and are in the units of the number of base substitutions per site (0.0001) shown in the bottom tree. This analysis involved 250 nucleotide sequences with a total of 29,420 positions in the final dataset, and all ambiguous positions were removed for each sequence pair (pairwise deletion option). The Delta variant taxa are indicated in the blue line, whereas the non-Delta variant taxa appeared in the green line. **(B)** A pie chart illustrates the proportion of Delta variant and non-Delta samples detected in the present study.

Phylogenetic analysis showed that about 69 samples (43%) of SARS-CoV-2 collected from Central Java and Yogyakarta provinces belonged to B.1.617.2 lineage (Delta variant), while 92 samples (57%) clustered in 14 different lineages based on the Pango nomenclature. They were B (1), B.1 (7), B.1.1 (2), B.1.459 (26), B.1.456 (1), B.1.462 (6), B.1.466.2 (21), B.1.468 (3), B.1.470 (11), B.1.570 (1), B.1.1.236 (1), B.1.36.19 (1), B.1.1.398 (3), and B.6 (1), and seven virus samples were not belonging to any of Pango lineages (“none”) (Figure 1). Except for the Delta variant, none of the virus samples collected from our study belonged to any VOC or variant of interest according to WHO labels for naming SARS-CoV-2 variants, including Alpha variant (B.1.7.7 + Q.x), which was first detected in Indonesia in January 2021. Delta variant (B.1.617.2 + AY.x) seemed to be the major VOC circulating in Indonesia, including in the Central Java and Yogyakarta provinces, from May 2021 up to now. Interestingly, we found that about 13% of virus samples from Central Java and Yogyakarta provinces were clustered into B.1.466.2 lineage (Figure 1) that is currently designated by WHO as a variant of alert for further monitoring.

### Characteristics of Patients With COVID-19

Most Delta variants (65.2%) detected in our study were cluster cases. The mean age of patients with Delta and the non-Delta variant was 27.3 ± 20.0 and 43.0 ± 20.9 (*p* = 3x10^−6^), respectively. The patients with the Delta variant consisted of 23 men and 46 women, while the patients with the non-Delta variant involved 56 men and 36 women (*p* = 0.001). The Ct value of the Delta variant (18.4 ± 2.9) was significantly lower than that of the non-Delta variant (19.5 ± 3.8) (*p* = 0.043) (Table 1).

**Table 1:** Characteristics of patients with COVID-19 from Yogyakarta and Central Java provinces, Indonesia.

**Characteristics**	**Total (*N* = 161)** ***N* (%);** **mean ± SD**	**Delta variant (*N* = 69) *N* (%);** **mean ± SD**	**Non-Delta variant** **(*N* = 92)** ***N* (%); mean ± SD**	***p*-value**
Ct value	19.1 ± 3.5	18.4 ± 2.9	19.5 ± 3.8	0.043^*^
Age (years)	36.3 ± 21.9	27.3 ± 20.0	43.0 ± 20.9	3 × 10^−6^^*^
•≥65	17 (10.6)	1 (1.4)	16 (17.4)	1 × 10^−6^^*^
• 18-<65	98 (60.9)	35 (50.7)	63 (68.5)	
• <18	46 (28.6)	33 (47.8)	13 (14.1)	
**Sex**				
• Male	79 (49.1)	23 (33.3)	56 (60.9)	0.001^*^
• Female	82 (50.9)	46 (66.7)	36 (39.1)	
**Comorbidities**				
• Obesity	10 (6.2)	3 (4.3)	7 (7.6)	0.51
• Diabetes	26 (16.1)	7 (10.1)	19 (20.7)	0.07
• Hypertension	22 (13.7)	4 (5.8)	18 (19.6)	0.012^*^
• Cardiovascular disease	15 (9.3)	4 (5.8)	11 (12)	0.18
• Chronic kidney disease	2 (1.2)	0	2 (2.2)	0.50
Smoking	5 (3.1)	1 (1.4)	4 (4.3)	0.39

* *Significant (p < 0.05); SD, standard deviation*.

### Association Between Independent Variables and COVID-19 Patients' Outcomes

Next, we associated the independent variables with the COVID-19 outcomes, hospitalization, and mortality. There were no significant differences in the hospitalization and mortality of patients with Delta and non-Delta variants (*p* = 0.80 and 0.29, respectively) (Table 2). None of the prognostic factors were associated with the hospitalization, except comorbidity of diabetes with the OR of 3.6 (95% CI = 1.02–12.5; *p* = 0.036) (Table 2). Moreover, the patients with the following factors have been associated with higher mortality rate than the patients without these factors: age ≥65 years, obesity, diabetes, hypertension, and cardiovascular disease with OR of 11 (95% CI = 3.4–36; *p* = 8 × 10^−5^), 27 (95% CI = 6.1–118; *p* = 1 × 10^−5^), 15.6 (95% CI = 5.3–46; *p* = 6 × 10^−7^), 12 (95% CI = 4–35.3; *p* = 1.2 × 10^−5^), and 6.8 (95% CI = 2.1–22.1; *p* = 0.003), respectively (Table 2).

**Table 2:** Association between independent variables and COVID-19 patients' outcomes.

**Variables**	**Hospitalized (*N*, %)**	***p*-value**	**OR (95% CI)**	**Mortality (*N*, %)**	***p*-value**	**OR (95% CI)**
**Delta variant**						
• Yes (*N* = 69)	50 (72.5)	0.80	1.1 (0.56–2.2)	6 (8.7)	0.29	0.57 (0.21–1.6)
• No (*N* = 92)	65 (70.7)			13 (14.1)		
**Age**						
•<65 (*N* = 98)	73 (74.5)	Ref	2.5 (0.5–12)	9 (9.2)	Ref	11 (3.4–36)
•≥65 (*N* = 17)	15 (88.2)	0.35	0.49 (0.23–1.02)	9 (52.9)	8 × 10^−5^*^^	0.22 (0.02–1.78)
•<18 (*N* = 46)	27 (58.7)	0.05		1 (2.2)	0.17	
**Sex**						
• Male (*N* = 79)	56 (70.9)	0.88	0.94 (0.47–1.8)	13 (16.5)	0.07	2.5 (0.9–6.9)
• Female (*N* = 82)	59 (72)			6 (7.3)		
**Comorbidity**						
**a. Obesity**						
Yes (*N* = 10)	10 (100)	0.06	9.26 (0.53–161.3)	7 (70)	1 × 10^−5^^*^	27 (6.1–118)
No (*N* = 151)	105 (69.5)			12 (7.9)		
**b. Diabetes**						
Yes (*N* = 26)	23 (88.5)	0.036^*^	3.6 (1.02–12.5)	12 (46.2)	6 × 10^−7^^*^	15.6 (5.3–46)
No (*N* = 135)	92 (68.1)			7 (5.2)		
**c. Hypertension**						
Yes (*N* = 22)	21 (95.5)	0.007^*^	10 (1.3–77)	10 (45.5)	1.2 × 10^−5^^*^	12 (4–35.3)
No (*N* = 139)	94 (67.6)			9 (6.5)		
**d. Cardiovascular disease**						
Yes (*N* = 15)	14 (93.3)	0.07	6.23 (0.79–48)	6 (40)	0.003^*^	6.8 (2.1–22.1)
No (*N* = 146)	101 (69.2)			13 (8.9)		
**e. Chronic kidney disease**						
Yes (*N* = 2)	2 (100)	1	2.05 (0.1–43.49)	1 (50)	0.22	7.8 (0.46–130)
No (*N* = 159)	113 (71.1)			18 (11.3)		
**Smoking**						
• Yes (*N* = 5)	2 (40)	0.14	0.25 (0.41–1.6)	1 (20)	0.47	1.9 (0.2–18)
• No (*N* = 156)	113 (72.4)			18 (11.5)		

* *Significant (p < 0.05); CI, confidence interval; OR, odds ratio*.

### Multivariate Analysis

Subsequently, we performed a multivariate analysis to find an independent factor affecting the COVID-19 outcomes. Multivariate analysis showed that age ≥65 years, obesity, diabetes, and hypertension were strong prognostic factors for the mortality of COVID-19 patients with the OR of 3.6 (95% CI = 0.58–21.9; *p* = 0.028), 16.6 (95% CI = 2.5–107.1; *p* = 0.003), 5.5 (95% CI=1.3–23.7; *p* = 0.021), and 5.8 (95% CI = 1.02–32.8; *p* = 0.047), respectively. In addition, no prognostic factors were associated with the hospitalization of patients with COVID-19 (Table 3).

**Table 3:** Multivariate analysis of the association between independent variables and COVID patients' outcomes.

**Variables**	**Hospitalized**		**Mortality**	
	**OR (95% CI)**	***p*-value**	**OR (95% CI)**	***p*–value**
Delta variant	1.3 (0.6–2.8)	0.47	3.6 (0.58–21.9)	0.167
Age (≥65 years)	0.7 (0.1–5.1)	0.79	11.5 (1.3–102.6)	0.028^*^
Sex (male)	0.9 (0.46–2.1)	0.98	3.2 (0.65–16)	0.15
**Comorbidity**				
• Obesity	–	0.99	16.6 (2.5–107.1)	0.003^*^
• Diabetes	1.68 (0.41–6.8)	0.46	5.5 (1.3–23.7)	0.021^*^
• Hypertension	10.4 (0.92–118)	0.058	5.8 (1.02–32.8)	0.047^*^
• Cardiovascular disease	3 (0.3–28.8)	0.32	0.7 (0.08–6.2)	0.75
• Chronic kidney disease	–	0.99	1.2 (0.015–96.6)	0.92
Smoking	0.11 (0.008–1.4)	0.09	0.5 (0.003–74.4)	0.78

* *Significant (p < 0.05); CI, confidence interval; OR, odds ratio; -, not applicable*.

## Discussion

We are able to show that the patients infected by the SARS-CoV-2 Delta variant have a significantly lower Ct value than the patients infected by the non-Delta variant. Our finding is compatible with previous studies (17, 31, 32). Our finding supports that the higher viral load of the Delta variant results in its characteristic of being more transmissible among humans (31, 33). It is associated with a higher reproductive number (R0) of the Delta variant (R0 = 7) than the parental SARS-CoV-2 and the Alpha variant (34). In addition, the Delta variant also has a significantly longer duration (18 days) of Ct value ≤30 than the original strain (13 days) (17). Again, this evidence implies higher transmissibility of the Delta variant than that of other strains.

In addition, it seems that the replication of SARS-CoV-2 is increased in a time-dependent manner during the early stage of infection. However, our study did not consider the interval days between the dates of the first infection and sample collection for the SARS-CoV-2 WGS. The SARS-CoV-2 WGS in our study was performed only based on the PCR's Ct value of ≤25 (22–24). These facts should be considered during the interpretation of our findings.

We also show that the Delta variant is not associated with the mortality and hospitalization of patients with COVID-19. Our findings are different from previous reports (15–17). Sheikh et al. (15) showed that the risk of hospitalization is two times higher in patients with Delta variant than in patients with the Alpha variant. Fisman et al. (16) revealed that the risk of hospitalization, admission of ICU, and mortality are significantly higher in the Delta variant than in N501Y-positive variants. They suggested that the Delta variant is more virulent than previous VOCs (16). Ong et al. (17) showed that the patients with the Delta variant were more severely affected with COVID-19 than the original variant. These differences might be due to differences in the host's genetic background (35). They identified a novel susceptibility locus for severe COVID-19 at the 3p21.31 gene cluster, consisting of *SLC6A20, LZTFL1, CCR9, FYCO1, CXCR6*, and *XCR1*, and their functions are related to COVID-19 (35). Further study is necessary to identify the genetic susceptibility locus for severe COVID-19 in our patients. Another difference between our study and previous reports is that we compared the outcomes of the Delta variant and non-VOC, while other reports compared the Delta variant and other VOCs [9; 10->15, 16]. Moreover, we focused on the impact of the Delta variant on COVID-19 outcomes, regardless of whether the Delta variants were originated from independent cases or clusters.

Interestingly, ~50% of Delta variant infected children, higher than non-Delta variant (~15%). Similar findings were reported by a previous study showing that the frequency of the Delta variant is higher in children aged 5–9 years than that of the non-Delta variant (15). While the Delta variant infected more women than men, the non-Delta variant infected more men than women. However, multivariate analysis did not show an association between sex and COVID-19 outcomes. It is similar to a study by Ong et al. (17). It should be noted that the impact of sex on the COVID-19 outcomes is still controversial (17, 18, 36, 37). While some studies showed that male has a higher risk for severe COVID-19 (18, 36), other reports do not support the association (17, 37).

Our findings reveal that older age and comorbidities, including obesity, diabetes, and hypertension, are independent prognostic factors for the mortality of patients with COVID-19. Our findings were similar to previous studies (18, 30, 36). Several mechanisms have been proposed for the increased risk of COVID-19 in patients with diabetes, including an elevated level of ACE-2 receptors and furin, and dysregulated immune response, while the following factors contribute for the obesity to be associated with the worse prognosis of COVID-19: the compromised ventilation at the base of the lung and immune response (38). The use of the antihypertension drug, particularly ACE-2 inhibitors and angiotensin receptor blockers, is associated with the upregulated expression of the ACE-2 receptor, resulting in a higher possibility of respiratory failure (38). Unfortunately, we do not have complete data on the use of ACE-2 inhibitors and angiotensin receptor blockers in our patients. Therefore, it is challenging to conclude that the increased mortality of COVID-19 in patients with hypertension is due to antihypertension.

Several studies have shown that the vaccinated individuals might have significantly less severe outcomes of COVID-19 if infected with the Delta variant (15, 39, 40). Unfortunately, we did not have complete data on the vaccination status of patients with COVID-19. In addition, some studies showed that different prevention and control measures might have a different impact on the outcomes of patients with COVID-19 (41–43). The government of Indonesia has also applied public health measures, i.e., mitigation intervention, including compulsory mask-wearing, personal protective equipment, social distancing measures, travel and mass gathering restrictions, quarantine of travelers arriving from overseas, isolation of confirmed cases and close contacts, contact tracing and testing, and school closures. However, our study did not consider the role of those interventions on the outcomes of patients with COVID-19.

Our cross-sectional design that srestricts the ability of causal inference, the wide variation of the 95% CI of ORs, and the lack of demographic information (including occupation, income, education level, low power of the study, and small sample size) were the weaknesses of our study. Moreover, we only determined the impact of the Delta variant and some comorbidities on the COVID-19 outcomes by overall means without considering other variables affecting the data, including vaccination history and public health measures.

## Conclusion

We show that the patients infected by the SARS-CoV-2 Delta variant have a lower Ct value than the patients infected by the non-Delta variant, implying that the Delta variant has a higher viral load, which might cause a more transmissible virus among humans. However, the Delta variant does not affect the COVID-19 outcomes in our patients. Our study also confirms that older age and comorbidity increase the mortality rate of patients with COVID-19.

## Data Availability Statement

The datasets of SARS-CoV-2 genomes presented in this study can be found in GISAID (https://www.gisaid.org/). The accession number(s) can be found in the Supplementary Table 1.

## Ethics Statement

The studies involving human participants were reviewed and approved by Faculty of Medicine, Public Health and Nursing, Universitas Gadjah Mada/Dr. Sardjito Hospital. Written informed consent to participate in this study was provided by the participants' legal guardian/next of kin.

## Author Contributions

G, KI, and NA conceived the study. G drafted the manuscript. MH, HW, and TW critically revised the manuscript for important intellectual content. M, DP, ITa, KV, MA, GG, LE, BN, EG, AD, AK, L, and SI performed the library preparation and NGS. G, MH, M, VS, Sl, ITr, ES, RK, KI, A, Si, I, NA, ED, DN, YP, SK, IL, NA, EA, TN, and TW collected the data. G, MH, HW, and M analyzed the data. All authors have read and approved the manuscript and agreed to be accountable for all aspects of the work in ensuring that questions related to the accuracy or integrity of any part of the work are appropriately investigated and resolved.

## Funding

This work was funded by the Ministry of Education, Culture, Research and Technology, Indonesia. The funders had no role in study design, data collection, and analysis, decision to publish, or manuscript preparation.

## Conflict of Interest

The authors declare that the research was conducted in the absence of any commercial or financial relationships that could be construed as a potential conflict of interest.

## Publisher's Note

All claims expressed in this article are solely those of the authors and do not necessarily represent those of their affiliated organizations, or those of the publisher, the editors and the reviewers. Any product that may be evaluated in this article, or claim that may be made by its manufacturer, is not guaranteed or endorsed by the publisher.
